# Rivaroxaban vs Vitamin K Antagonist in Patients With Atrial Fibrillation and Advanced Chronic Kidney Disease

**DOI:** 10.1016/j.jacadv.2023.100813

**Published:** 2024-01-05

**Authors:** Reinhold Kreutz, Gilbert Deray, Jürgen Floege, Marianne Gwechenberger, Kai Hahn, Andreas R. Luft, Pontus Persson, Christoph Axthelm, Juerg Hans Beer, Jutta Bergler-Klein, Nicolas Lellouche, Jens Taggeselle, Craig I. Coleman, Jan Beyer-Westendorf, XARENO registry, Albano Laetitia, Albert Catherine, Alexandre Joachim, Al-Zoebi Ayham, Annweiler Cedric, Auer Johann, Balgobin Sanjeet, Beige Joachim, Berami Ahmed, Berneau Jean-Baptiste, Biggar Patrick, Birkemeyer Ralf, Bondke Christina, Bonin-Schnabel Renate, Bonnemeier Hendrik, Bouiller Marc, Boureau Anne-Sophie, Brachmann Johannes, Brosche Jörg, Caudmont Sebastien, Cayla Guillaume, Charpy Vianney, Constans Joel, Dally Jean-Baptiste, De Geeter Guillaume, Debelle Fédéric, Decoulx Eric, Delarche Nicolas, Delle Karth Georg, Delsart Pascal, Derndorfer Michael, Desprets Laurent, Dillinger Jean-Guillaume, Dubart Camille, Eberhard Katrin, Eichinger-Hasenauer Sabine, Eissing Volker, Erley Christiane, Esteve Jean-Baptiste, Ferrari Emile, Fossey-Diaz Virginie, Fromentin Stéphane, Gallouj Karim, Gandjbakhch Estelle, Garnier Anne-Sophie, Gilis Laure, Gondouin Bertrand, Grundmann Franziska, Gueffet Isabelle, Haaß Sebastian, Haguenhauer Didier, Hannedouche Thierry, Häusler Karl Georg, Heinz Gerd-Ulrich, Herold Philipp, Hertting Klaus, Hoffer Etienne, Hoyer Joachim, Hügl Burkhard, Jänsch Sybille, Jean-Louis Georges, Jeserich Michael, Jung Werner, Kassis Samuel, Kellner Bernd-Thomas, Ketteler Marcus, Kielstein Jan Thomas, Koning René, Krämer Fabian, Krzesinski Jean-Marie, Lammers Ulrich, Lefebvre Jean-Marie, Legrand Eric, Leschke Matthias, Lodde Bernhard-Paul, Maalouli Christian, Mahnkopf Christian, Mailliez Sebastien, Mansourati Jacques, Marijon Eloi, Meyer Christian, Moll Detlev, Montalescot Gilles, Motte Serge, Mouquet Vincent, Nedeltchev Krassen, Neykova Anna, Nothroff Jörg, Poyet Raphael, Prondzinsky Roland, Rauch-Kröhnert Ursula, Richard Frank, Rieker Werner, Rocco Andrea, Rostock Thomas, Scherr Daniel, Schlitt Axel, Schmidt-Gürtler Hans, Schön Norbert, Schwab Johannes, Schwencke Carsten, Schwimmbeck Peter, Schwinger Robert H. G, Schwittay Andreas, Sibon Igor, Spengler Ulrike, Stadelmann Alexander, Steinwender Clemens, Stöhring Reinhard, Stolear Jean-Claude, Taldir Guillaume, Tartière Jean-Michel, Treille Serge, Tremolieres Pierre, Tubail Zead, Warling Xavier, Wetzstein Morgane, Zaman Adrian, Zemmrich Claudia

**Affiliations:** a Charité - Universitätsmedizin Berlin, Institute of Clinical Pharmacology and Toxicology, Berlin, Germany; b Department of Nephrology, Pitié-Salpêtrière Hospital, Paris, France; c Division of Nephrology and Clinical Immunology, RWTH Aachen University Hospital, Aachen, Germany; d Division of Cardiology, University Department of Internal Medicine II, Medical University of Vienna, Vienna, Austria; e Nephrologische Praxis, Dortmund, Germany; f Klinik für Neurologie, Universitätsspital Zürich, Zürich, Switzerland; g Cereneo, Center for Neurology and Rehabilitation, Vitznau, Switzerland; h Institut für Vegetative Physiologie, Charité - Universitätsmedizin Berlin, Berlin, Germany; i Cardiologicum Pirna & Dresden, Dresden, Germany; j Department Innere Medizin, Kantonsspital Baden, Baden, Switzerland; k Center of Molecular Cardiology, University of Zürich, Zürich, Switzerland; l Service de Cardiologie 1, Centre Hospitalier Universitaire Henri Mondor, Créteil, France; m Praxis Dr med. Jens Taggeselle, Markkleeberg, Germany; n Department of Pharmacy Practice, University of Connecticut School of Pharmacy, Storrs, Connecticut, USA; o Evidence-Based Practice Center, Hartford Hospital, Hartford, Connecticut, USA; p Department of Medicine I, Division Thrombosis & Hemostasis, University Hospital Carl Gustav Carus Dresden, Dresden, Germany

**Keywords:** bleeding, elderly, kidney failure, oral anticoagulation

## Abstract

**Background:**

Treatment with vitamin K antagonists (VKAs) has been linked to worsening of kidney function in patients with atrial fibrillation (AF).

**Objectives:**

XARENO (Factor XA-inhibition in RENal patients with non-valvular atrial fibrillation Observational registry; NCT02663076) is a prospective observational study comparing adverse kidney outcomes in patients with AF and advanced chronic kidney disease receiving rivaroxaban or VKA.

**Methods:**

Patients with AF and an estimated glomerular filtration rate (eGFR) of 15 to 49 mL/min/1.73 m^2^ were included. Blinded adjudicated outcome analysis evaluated adverse kidney outcomes (a composite of eGFR decline to <15 mL/min/1.73 m^2^, need for chronic kidney replacement therapy, or development of acute kidney injury). A composite net clinical benefit outcome (stroke or systemic embolism, major bleeding, myocardial infarction, acute coronary syndrome, or cardiovascular death) was also analyzed. HRs with 95% CIs were calculated using propensity score overlap weighting Cox regression.

**Results:**

There were 1,455 patients (764 rivaroxaban; 691 VKA; mean age 78 years; 44% females). The mean eGFR was 37.1 ± 9.0 in those receiving rivaroxaban and 36.4 ± 10.1 mL/min/1.73 m^2^ in those receiving VKA. After a median follow-up of 2.1 years, rivaroxaban was associated with less adverse kidney outcomes (HR: 0.62; 95% CI: 0.43-0.88) and all-cause death (HR: 0.76, 95% CI: 0.59-0.98). No significant differences were observed in net clinical benefit.

**Conclusions:**

In patients with AF and advanced chronic kidney disease, those receiving rivaroxaban had less adverse kidney events and lower all-cause mortality compared to those receiving VKA, supporting the use of rivaroxaban in this high-risk group of patients.

The prevalence of both atrial fibrillation (AF) and chronic kidney disease (CKD) increases with advanced age and these patients are at increased risk for both thrombotic and bleeding events.^1^^,^^2^ Nevertheless, long-term oral anticoagulation (OAC) is utilized in most patients with AF and CKD to prevent thromboembolic stroke and systemic embolism. As CKD increases bleeding risk, patients with AF and advanced CKD stages ≥4 represent a high-risk population for which treatment decisions for or against OAC are especially challenging.^3^^,^^4^

OAC with vitamin K antagonists (VKAs) has been associated with accelerated calcification of coronary and extra-coronary arteries^5^^,^^6^ as well as cardiac valves.^7^^,^^8^ A systematic review and meta-analysis revealed a significantly elevated OR (1.8, IQR: 1.43-2.24) for extra-coronary calcifications in patients treated with a VKA as compared to patients without anticoagulation or receiving other anticoagulants.^9^ The observed accelerated decline of kidney function in patients with AF and CKD treated with VKA^10^ has also been attributed to these vascular side effects of VKA. However, there are additional concerns about the risk of anticoagulation nephropathy^11^^,^^12^ during VKA treatment, although this condition may develop in response to any anticoagulant.^13^ It has been specifically associated with overdosing of VKA with international normalized ratio levels above the therapeutic range.^10^, ^11^, ^12^ However, studies have demonstrated that, compared to VKA, OAC with direct oral anticoagulants (DOACs) has less impact on renal function decline.^14^, ^15^, ^16^, ^17^, ^18^, ^19^ DOACs including rivaroxaban may not only lack a detrimental effect on arterial calcification but may even induce kidney sparing or preserving effects attributable to inhibition of protease-activated receptor-mediated pro-inflammatory effects in the vasculature^20^ and in early vascular aging in CKD.^21^

Available large registries assessing the effectiveness and safety of OAC in patients with AF included only a small proportion of patients with concomitant advanced CKD. Hence, in the large GARFIELD-AF (Global Anticoagulant Registry in the Field-Atrial Fibrillation) registry, physicians classified 10.9% of patients as having moderate-to-severe CKD,^22^ while only a minor group (1.7%) of the overall population (564 from 33,024 patients) were diagnosed with advanced CKD 4 or 5 (ie, with estimated glomerular filtration rates [eGFRs] <30 mL/min/1.73 m^2^). Similarly, the large PREFER (PREvention oF Thromboembolic Events–European Registry in Atrial Fibrillation) multicenter registry of 6,412 AF patients included only a small portion of 842 (13.1%) patients with chronic kidney failure, while CKD stages were not further classified.^3^ The XARENO registry (Factor XA-inhibition in RENal patients with non-valvular atrial fibrillation Observational registry) is the first prospective registry specifically focused on AF patients with advanced stages of CKD receiving rivaroxaban or VKA with an adjudicated blinded analyses of outcomes.

## Methods

### Study design

XARENO was an investigator-initiated, multicenter, prospective, noninterventional study conducted in Europe (Germany, Austria, Switzerland, France, Belgium, and Luxembourg).^23^ Management of patients was at the discretion of the participating physicians. The study was registered with clinical trials.gov (NCT02663076).

Inclusion criteria were a diagnosis of AF as diagnosed by the participating physicians, adult age (≥18 years), and an eGFR between 15 and 49 mL/min/1.73 m^2^ as estimated by the Chronic Kidney Disease Epidemiology Collaboration equation^24^ and an indication for anticoagulation. The XARENO protocol was approved by all responsible independent ethic committees and informed consent was obtained for all recruited patients. The study protocol has been reported (Supplemental Appendix).^23^ To be included, patients had to be treated with rivaroxaban or VKA for at least 3 months prior to enrollment. Patients continued their ongoing anticoagulation treatment regimen when consented into XARENO (Supplemental Figure 1). Prespecified follow-up was at least 12 months followed by a planned extended data collection period for 1 up to 2 additional years.

### Outcomes

The primary outcome of interest was the absolute change in eGFR in mL/min/1.73 m^2^ (as estimated by the Chronic Kidney Disease Epidemiology Collaboration equation)^24^ at 12 months. Clinical outcomes including any adverse kidney outcome, a composite of chronic kidney replacement therapy (KRT), an eGFR <15 ml/min/1.73 m^2^, or acute kidney injury, net clinical benefit, a composite of stroke or other thromboembolic events, major bleeding, and all-cause mortality, and each composite’s individual component. Baseline characteristics were analyzed using descriptive statistics. Categorical variables were reported as percentages and continuous variables as mean ± SD or median (IQR), where appropriate.

### Propensity score overlap weighting analysis

To adjust for imbalances in patient characteristics between the rivaroxaban and VKA arms at baseline, we calculated propensity scores^25^ based upon multivariable logistic regression using 42 distinct demographic, comorbidity, laboratory, and concurrent medication variables known to be risk factors for differential OAC exposure (Table 1, Supplemental Appendix). Estimated propensity scores were subsequently used to weight patients for analysis using overlap weighting (OLW).^26^ Propensity score OLW assigns weights to patients proportional to their probability of belonging to the opposing treatment cohort (ie, rivaroxaban patients were weighted by the probability of receiving VKA (or 1—the propensity score) and VKA patients were weighted by the probability of receiving rivaroxaban (the propensity score). OWL was chosen as the primary method for confounder adjustment because it allows for all eligible patients to be included in the analysis (unlike propensity score matching which typically results in sample size reduction in one or both cohorts), it assigns greater weight to patients in which treatment cannot be predicted and lesser weight to patients with extreme propensity scores preventing outliers from dominating the analysis and decreasing precision (a concern with inverse probability weighting) and because overlap weighting has the favorable property of resulting in the exact balance (absolute standardized differences [ASDs] = 0%) for all variables included in the multivariable logistic regression model used to derive propensity scores.

**Table 1:** Patient Characteristics Prior to and Following Propensity Score Overlap Weighting

	Prior OLW			Following OLW		
	Rivaroxaban (n = 764)	VKA (n = 691)	ASD, %	Rivaroxaban (n = 764)	VKA (n = 691)	ASD, %
Demographics						
Age, y	77.7 ± 7.4	78.5 ± 7.6	**10.7**	78.2 ± 7.2	78.2 ± 7.5	0
Age ≥75 y	70.7%	74.7%	9.0	73.2	73.2	0
Male	54.3%	57.5%	6.3	56.3	56.3	0
Ethnicity, White	98.4%	99.0%	4.9	99.0	99.0	0
AF type						
Persistent/permanent	48.4%	57.0%	**17.3**	54.2	54.2	0
Paroxysmal	48.7%	41.5%	**14.4**	43.9	43.9	0
Unknown	2.9%	1.4%	9.9	2.0	2.0	0
Time since AF diagnosis						
<2 y	34.2%	21.0%	**29.8**	26.5	26.5	0
2 to <5 y	32.1%	34.2%	4.4	32.5	32.5	0
≥5 y	31.8%	42.7%	**22.7**	38.7	38.7	0
Unknown	2.0%	2.2%	1.5	2.3	2.3	0
Time since CKD diagnosis						
<2 y	55.2%	44.6%	**21.4**	50.0	50.0	0
≥2 y	42.3%	52.8%	**21.2**	47.4	47.4	0
Country						
Germany	61.9%	56.4%	**11.1**	61.9	61.9	0
France	24.2%	30.8%	**14.8**	25.8	25.8	0
Austria	8.5%	6.9%	5.8	7.0	7.0	0
Belgium	4.1%	3.2%	4.7	3.3	3.3	0
Switzerland	1.3%	2.6%	9.4	2.0	2.0	0
Unknown	2.5%	2.6%	0.7	2.6	2.6	0
Alcohol use						
None	75.1%	76.3%	2.6	76.4	76.4	0
Yes	17.8%	19.4%	4.1	18.3	18.3	0
Unknown	7.1%	4.3%	**11.8**	5.3	5.3	0
Smoker						
Never	65.1%	63.2%	3.8	64.6	64.6	0
Former	28.0%	31.3%	7.1	29.1	29.1	0
Current	3.3%	3.2%	0.5	3.3	3.3	0
Unknown	3.7%	2.3%	7.9	3.0	3.0	0
BMI, kg/m^2^						
<30	58.6%	62.7%	8.2	61.6	61.6	0
30-39.9	36.3%	31.5%	**10.0**	32.8	32.8	0
≥40	3.7%	4.6%	4.8	4.3	4.3	0
Unknown	1.4%	1.2%	2.5	1.3	1.3	0
eGFR, mL/min/1.73 m^2^						
<15	0.1%	1.3%	**13.9**	0.3	0.3	0
15-19.9	1.7%	8.1%	**30.0**	3.3	3.3	0
20-29.9	11.1%	26.5%	**40.1**	17.5	17.5	0
30-39.9	33.1%	25.6%	**16.5**	31.1	31.1	0
40-49.9	35.2%	21.7%	**30.3**	29.1	29.1	0
≥50	7.1%	3.9%	**13.9**	5.0	5.0	0
Unknown	11.6%	12.9%	3.8	13.6	13.6	0
Comorbidities						
Hypertension	79.7%	80.6%	2.2	79.8	79.8	0
Diabetes	39.3%	41.7%	4.9	40.9	40.9	0
Any coronary artery disease	28.0%	32.1%	9.0	30.5	30.5	0
Myocardial infarction	11.9%	14.3%	7.2	11.6	11.6	0
Percutaneous coronary intervention	16.0%	18.1%	5.6	17.2	17.2	0
Coronary bypass grafting	7.1%	10.7%	**12.8**	9.3	9.3	0
Ischemic stroke	8.2%	7.1%	4.3	7.3	7.3	0
Intracranial hemorrhage	0.4%	0.7%	4.4	0.7	0.7	0
Heart failure	21.7%	22.7%	2.4	22.5	22.5	0
Peripheral artery disease	8.8%	9.1%	1.2	8.6	8.6	0
Prior venous thromboembolism	5.9%	8.4%	9.7	7.3	7.3	0
Chronic lung disease	11.0%	10.4%	1.9	11.0	11.0	0
Cancer	12.2%	9.7%	7.9	10.6	10.6	0
Anemia	3.8%	7.4%	**15.7**	5.0	5.0	0
Liver dysfunction	3.3%	4.3%	5.6	4.0	4.0	0
Osteoporosis	2.7%	2.2%	3.7	2.3	2.3	0
Comedications						
Angiotensin-converting enzyme inhibitor or angiotensin receptor blocker	69.9%	66.3%	7.8	69.2	69.2	0
Beta-blocker	78.3%	79.7%	3.6	79.7	79.7	0
Calcium-channel blocker	31.9%	30.1%	4.0	31.6	31.6	0
Diuretic	76.4%	85.4%	**22.9**	82.7	82.7	0
Amiodarone	21.1%	19.7%	3.5	20.3	20.3	0
Other antiarrhythmic medication	6.8%	3.6%	**14.4**	4.6	4.6	0
Statin	54.5%	56.9%	4.9	55.8	55.8	0
Low-dose aspirin	7.9%	11.4%	**12.1**	9.6	9.6	0
P2Y12 inhibitor	8.1%	6.4%	6.7	7.3	7.3	0
Nonsteroidal anti-inflammatory	5.0%	5.5%	2.4	5.3	5.3	0
Insulin	19.2%	20.1%	2.2	19.6	19.6	0
Other diabetic medication	22.1%	21.1%	2.4	22.2	22.2	0
Erythropoietin	1.6%	4.8%	**18.4**	2.3	2.3	0
Vitamin D supplementation	18.3%	23.3%	**12.3**	19.9	19.9	0

Values are mean ± SD or %.

ASD values of at least 10% are given in **bold**.

AF = atrial fibrillation; ASD = absolute standardized difference; BMI = body mass index; CKD = chronic kidney disease; eGFR = estimated glomerular filtration rate; OLW = overlap weighting; VKA = vitamin K antagonist.

### Statistical analysis

All analyses used the intention-to-treat dataset. The difference in mean change in eGFR from baseline between groups was compared using a Student’s *t*-test. Clinical outcomes were compared between groups using a propensity score OWL Cox proportional hazards regression model using a robust estimator. Patients were censored in the Cox models at the time of outcome occurrence, death, or end of study follow-up. Results are presented as HRs and corresponding 95% CIs. The proportionality assumption was assessed by evaluating Schoenfeld residuals and was not significant for any outcome. As a post hoc sensitivity analysis, we constructed a Fine and Gray competing risk regression model for the outcome of any adverse kidney outcome controlling for death. All database management and statistical analysis were performed using SAS version 9.4 (SAS Institute). No statistical adjustments for multiple hypothesis testing were performed. The modest sample size precluded meaningful subgroup analyses according to patient characteristics. A strength of the study was its prospective design with blinded adjudicated outcome analyses.

## Results

### Patient characteristics before and after OLW

**Figure 1:**
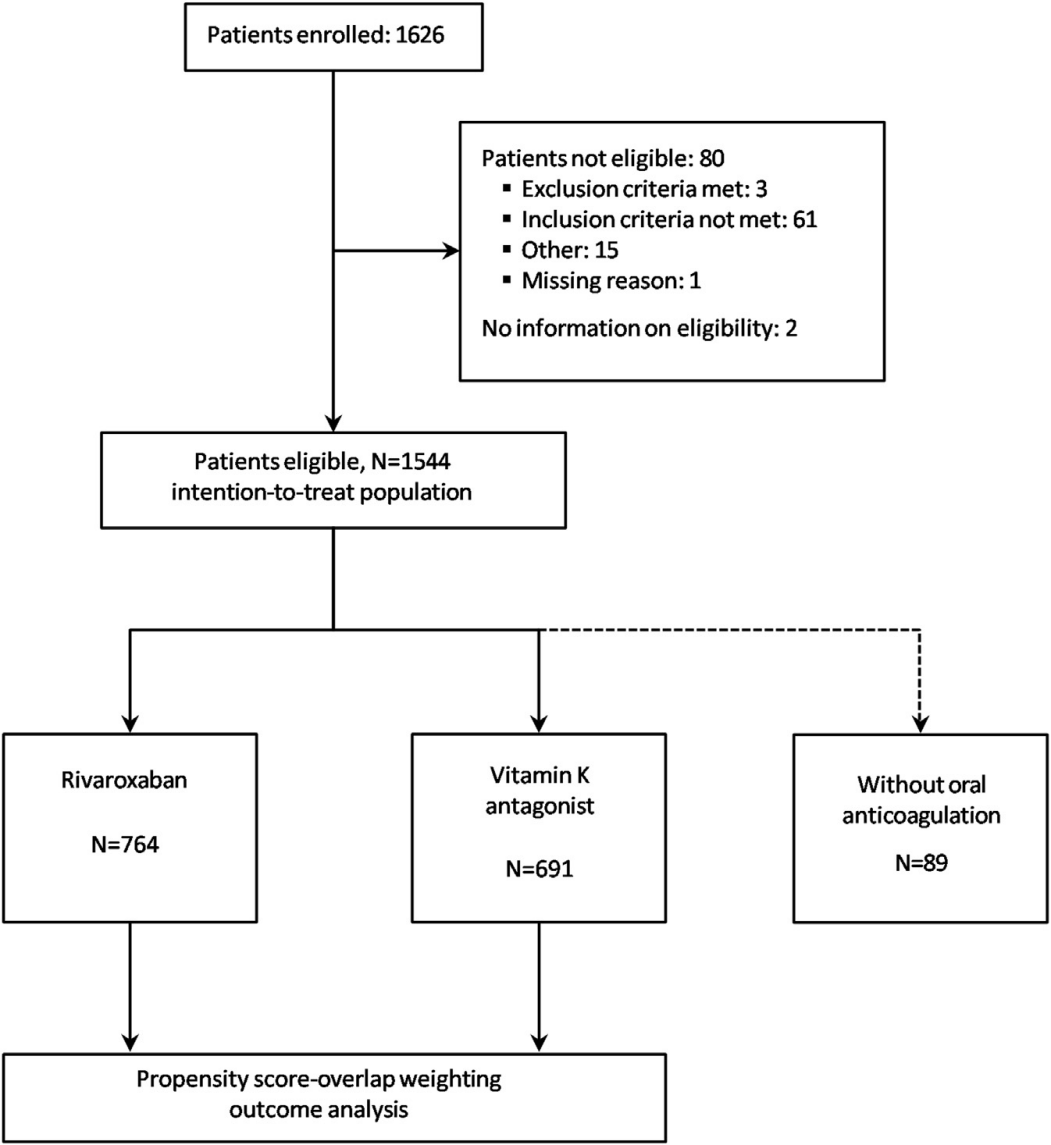
**Flow Diagram With Patient Disposition in the XARENO Registry** The flow of patients who participated in the registry is shown, including the number originally enrolled, and eligible participants included into the intention-to-treat population. The small group of 89 patients that were not treated with anticoagulation at the discretion of participating physicians were according to the study protocol^23^ not included in the propensity score overlap weighting outcome analysis. XARENO = Factor XA-inhibition in RENal patients with non-valvular atrial fibrillation Observational registry.

Overall 1,455 patients receiving either rivaroxaban (N = 764) or a VKA (N = 691) were included in the study and available for propensity score OLW outcome analysis (Figure 1). Patient characteristics including demographics, comorbidities, and comedications prior and following OLW are summarized in Table 1. Patients in rivaroxaban and VKA arms differed as evidenced by multiple variables with an ASD >10% prior to OLW, while patient characteristics were well-balanced (ASD = 0%) for all covariates entered in the propensity score model after OLW.

Prior to OLW, the mean age of patients in the VKA group was 78.5 ± 7.6 years and somewhat higher as compared to rivaroxaban patients 77.7 ± 7.4 years. At baseline, 94% of patients had eGFR values below 45 mL/min/1.73 m^2^, that is, were in CKD stage ≥3b. Mean eGFR after OLW at baseline was 37.1 ± 9.0 in the rivaroxaban and 36.4 ± 10.1 mL/min/1.73 m^2^ in the VKA group. After 12 months, the mean eGFR change from baseline in the rivaroxaban group was +0.46 ± 9.46 mL/min/1.73 m^2^ and +0.27 ± 8.66 mL/min/1.73 m^2^ in the VKA group (group difference 0.19; 95% CI: −1.70 to 2.08 mL/min/1.73 m^2^; *P* = 0.85). Following OLW, the distribution of comorbidities including hypertension, diabetes, myocardial infarction, heart failure, and a history of stroke was similar between groups. Accordingly, the median CHA_2_DS_2_-VASc scores were 4 (IQR: 3-5) in both rivaroxaban and VKA groups. The median mHAS-BLED scores were 2 (IQR: 1-2) in both groups. The median time of follow-up was 757 (IQR: 444-1,043) days for the total study population and 777 (IQR: 497-1,083) days for rivaroxaban vs 745 (IQR: 389-1,022) days for VKA.

### Medication use

Of the 764 patients who received rivaroxaban, 610 patients (79.8%) received the 15 mg and 117 patients (15.3%) the 20 mg dose (Table 2). Different VKA agents were used in the VKA group, reflecting known differences in clinical practice in participation countries. The median (25%, 75% range) time-in-therapeutic range for the 691 VKA patients was 62.6% (35.7%, 82.0%). The use of any antiplatelet drugs (either low-dose aspirin or a P2Y12 inhibitor was similar between groups.

**Table 2:** Oral Anticoagulant Treatment

	Rivaroxaban (n = 764)	Vitamin K Antagonist (n = 691)
Rivaroxaban dose		NA
15 mg	610 (79.85)	
20 mg	117 (15.3)	
VKA type	NA	
Acenocoumarol		26 (3.8)
Fluindione		124 (17.9)
Phenprocoumon		452 (65.4)
Warfarin		89 (12.9)

Values are n (%).

NA = not applicable; VKA = vitamin K antagonist.

### Outcomes

**Figure 2:**
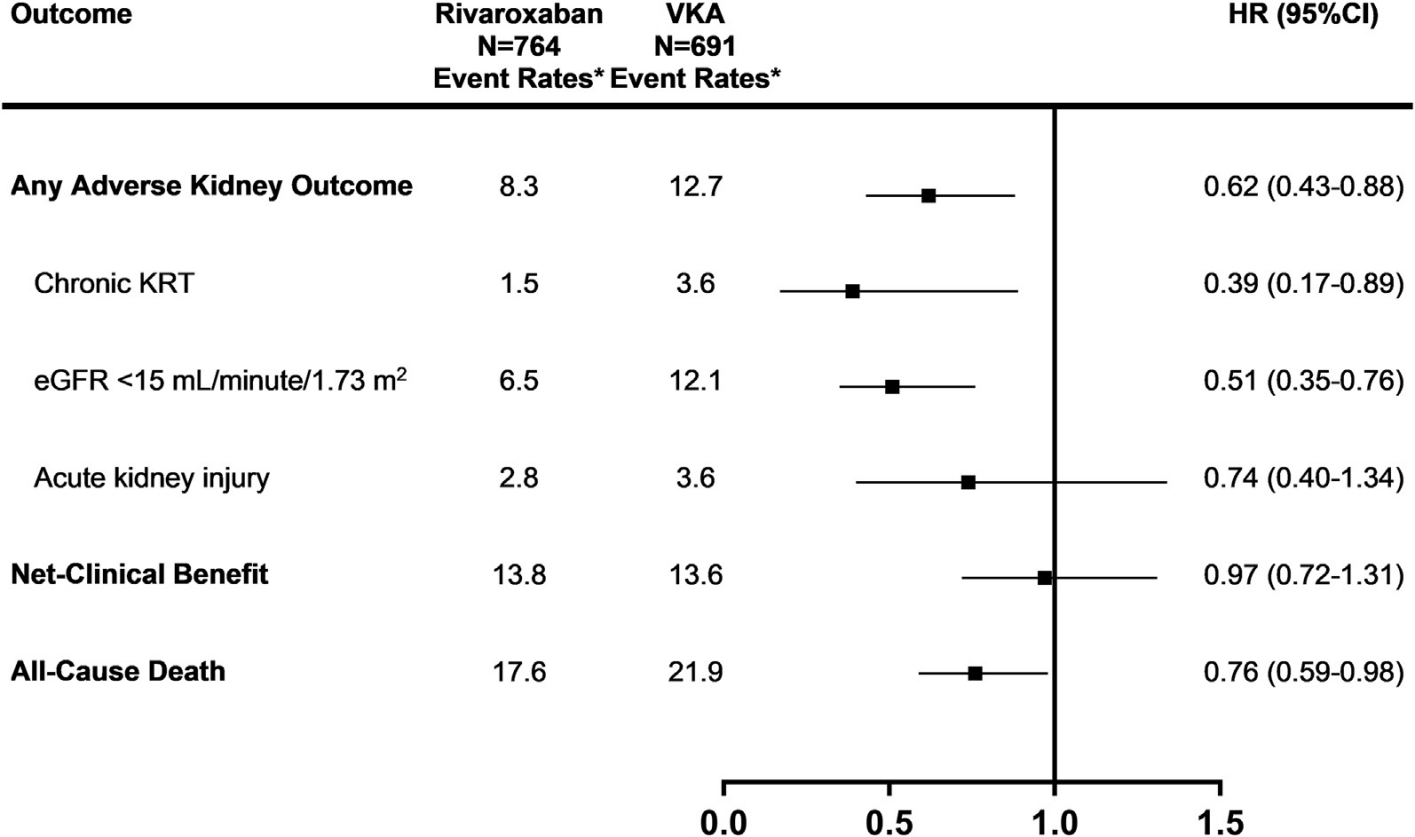
**Main Outcomes** Annual event rates, and adjusted HR (95% CI) for the comparison between rivaroxaban and vitamin K antagonist (VKA) after propensity score overlap weighting outcome analysis. ∗Event rates per 100 patient-years. eGFR = estimated glomerular filtration rate; KRT = kidney replacement therapy.

Weighted Cox regression analyses showed that rivaroxaban as compared to VKA was associated with significant reductions in adverse kidney outcomes (Table 3) including a significant 38% hazard reduction for the composite of any adverse kidney outcome (HR: 0.62; 95% CI: 0.43-0.88), a 61% reduction in the need for chronic KRT (HR: 0.39; 95% CI: 0.17-0.89) (Central Illustration), and a 49% reduction in the hazard of renal decline to an eGFR <15 mL/min/1.73 m^2^ (HR: 0.51; 95% CI: 0.35-0.76) (Table 3, Figure 2). No significant difference in acute kidney injury was observed between the rivaroxaban and VKA arms (HR: 0.74; 95% CI: 0.40-1.34). Similarly, no significant differences were found for the composite net clinical benefit outcome (HR: 0.97; 95% CI: 0.72-1.31) or any of the individual component outcomes including no significant difference between groups for cardiovascular death (HR: 0.82; 95% CI: 0.54-1.25) (Table 3). However, all-cause mortality was found to be reduced with rivaroxaban compared to VKA (HR: 0.76; 95% CI: 0.59-0.98) (Table 3, Figure 2, Central Illustration). Taking rivaroxaban on the initial study visit was associated with a 24% lower relative hazard of discontinuing the anticoagulation therapy compared to VKA (HR: 0.76; 95% CI: 0.62-0.92) (Table 3). Our sensitivity analysis showed that the hazard for the composite of any adverse kidney outcome with death as a competing risk was similar to the main finding (sub-HR: 0.60; 95% CI: 0.36-0.99).

**Table 3:** Propensity Score Overlap-Weighted Outcomes

	Rivaroxaban (n = 764)	VKA (n = 691)	HR (95% CI)
Any adverse kidney outcome	8.3	12.7	0.62 (0.43-0.88), *P* = 0.01
Chronic kidney replacement therapy^a^	1.5	3.6	0.39 (0.17-0.89), *P* = 0.03
eGFR <15 mL/min/1.73 m^2^^a^	6.5	12.1	0.51 (0.35-0.76), *P* = 0.001
Acute kidney injury^a^	2.8	3.6	0.74 (0.40-1.34), *P* = 0.32
Net clinical benefit	13.8	13.6	0.97 (0.72-1.31), *P* = 0.84
Stroke/systemic embolism or cardiovascular death	8.0	8.8	0.88 (0.61-1.29), *P* = 0.52
Stroke/systemic embolism^b^	1.7	1.4	1.19 (0.50-2.79), *P* = 0.69
Stroke/TIA/systemic embolism	2.3	2.4	0.92 (0.44-1.94), *P* = 0.83
Cardiovascular death^b^	6.4	7.5	0.82 (0.54-1.25), *P* = 0.36
Myocardial infarction or acute coronary syndrome^b^	2.3	1.3	1.68 (0.68-4.13), *P* = 0.26
Major bleeding^b^	5.3	4.9	1.05 (0.65-1.71), *P* = 0.84
All-cause death	17.6	21.9	0.76 (0.59-0.98), *P* = 0.03
Initial anticoagulant discontinuation	32.4	39.6	0.76 (0.62-0.92), *P* = 0.005

Values are %/y unless otherwise indicated.

Annual event rates and adjusted HRs with 95% CIs for the comparison between rivaroxaban and vitamin K antagonist (VKA) after propensity score overlap weighting outcome analysis.

eGFR = estimated glomerular filtration rate; TIA = transient ischemic attack.

a Individual components of any adverse kidney outcome.

b Individual components of net clinical benefit.

**Central Illustration:**
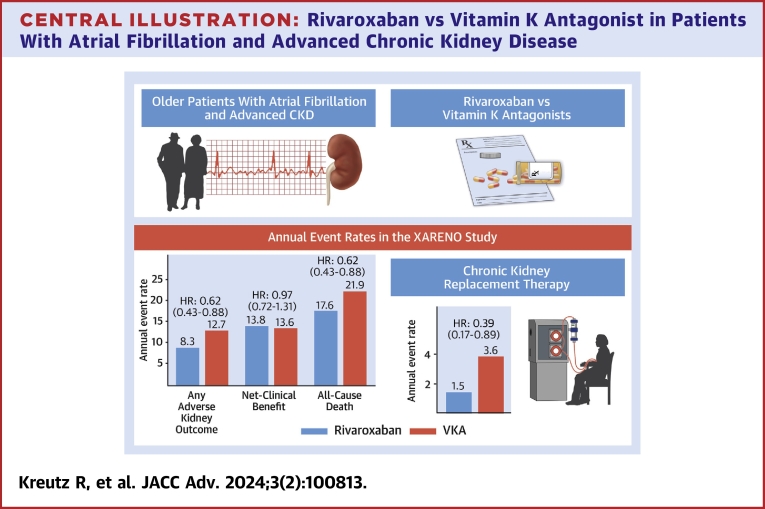
**Rivaroxaban vs Vitamin K Antagonist in Patients With Atrial Fibrillation and Advanced Chronic Kidney Disease** Annual event rates and adjusted HRs (95% CI) for the comparison between rivaroxaban and vitamin K antagonist (VKA) after propensity score overlap-weighted outcome analysis. CKD = chronic kidney disease; XARENO = Factor XA-inhibition in RENal patients with non-valvular atrial fibrillation Observational registry.

## Discussion

### Kidney outcomes

In the current study, rivaroxaban use in AF patients with comorbid CKD was associated with a 38% reduction in the hazard of experiencing an adverse kidney outcome, including a 61% reduction in need for chronic KRT and a 49% reduction in progression of kidney function decline to an eGFR <15 mL/min/1.73 m^2^ vs a VKA. The kidney benefits of rivaroxaban vs VKA observed in XARENO are consistent with previous retrospective real-world database studies.^15^, ^16^, ^17^ It is unclear whether the higher risk of adverse kidney outcomes with VKA compared to rivaroxaban observed in the current and prior real-world evidence studies^15^, ^16^, ^17^ is due to a detrimental effect of VKAs on vascular injury and calcification,^5^^,^^9^^,^^27^ kidney sparing or preservation effects of rivaroxaban possibly attributable to reduced protease-activated receptor-mediated inflammation^20^^,^^27^ or some combination of both.

### Thromboembolic and bleeding outcomes

In respect to thrombotic and major bleeding outcomes, no significant difference in the composite net clinical benefit outcome or any of the individual component outcomes was observed when comparing rivaroxaban and VKA in agreement with the ROCKET–AF (Rivaroxaban Once-daily, oral, direct factor Xa inhibition compared with vitamin K antagonism for prevention of stroke and Embolism Trial in Atrial Fibrillation) randomized controlled trial.^28^

XARENO did not aim and was not powered to show reductions in thrombotic or bleeding outcomes in patients with advanced CKD.^23^ However, a recent patient-level network meta-analysis of the RCTs that compared DOACs with warfarin indicated that, compared with warfarin, standard-dose DOAC use was more effective to reduce the risk for stroke/systemic embolism across the spectrum of kidney function above CrCl of at least 25 mL/min.^29^ Another recent network meta-analysis in patients with AF and CKD (overall 19 studies)^30^ including both subgroup analysis data from these RCTs (5 studies) and observational studies (14 studies) found that all 4 DOACs (apixaban, dabigatran, edoxaban, and rivaroxaban) led to significant risk reductions for stroke or thromboembolism as compared to warfarin in CKD patients. Additionally, except for dabigatran, all 3 Factor Xa inhibitors reduced the risk of major bleeding. For separate analysis in advanced CKD with CrCl below 30 ml/min, only 5 studies were available for inclusion and among DOAC patients those specified as rivaroxaban users dominated the analysis (n = 2,677) over apixaban users (n = 135). While hazards were numerically lower for both rivaroxaban and apixaban no significant risk reduction in stroke or thromboembolism was observed in comparison to warfarin. Kidney outcomes were not considered in this network meta-analysis.^30^

### Patients of older age with AF and advanced CKD

Of interest, a randomized controlled trial in patients with AF aged 80 years or older (mean age 86.6 years) included also patients with advanced CKD (inclusion criterion CrCl ≥15 mL/min; mean CrCl 36.3 mL/min).^31^ In this trial, active low-dose treatment with edoxaban 15 mg once daily, a dose which is currently not approved for clinical use as monotherapy in AF, was compared to placebo in overall 984 patients of whom 681 patients completed the trial.^31^ OAC resulted in a significant risk reduction (HR: 0.34; 95% CI: 0.19-0.61; *P* < 0.001) in the rate of stroke and systemic embolism as compared to placebo, while the risk for major bleeding was numerically but not statistically significantly higher (HR: 1.87; 95% CI: 0.90-3.89; *P* = 0.09) in the OAC group and death rates were similar between groups.^31^ However, the question whether low-dose treatment with the Factor Xa inhibitor edoxaban as compared to patients without OAC would impact on kidney outcomes was not addressed in this study.^31^

Annual mortality was high in the XARENO study population which is in agreement with the mean age of about 78 years and the inclusion of a high-risk AF population in which the presence of advanced CKD contributed further to mortality.^32^ All-cause mortality was found to be reduced with rivaroxaban compared to VKA, but cardiovascular mortality was not. Whether the reduction in all-cause mortality was a downstream effect of slowing the decline in kidney function or due to residual confounding specific to all-cause mortality or noncardiovascular death (∼7 of 10 AF patients die of cardiovascular causes)^33^ in XARENO is unclear. Finally, rivaroxaban was associated with better persistence to therapy than VKA; a finding that mirrors observations from prior real-world studies.^34^

### Study limitations

This study has limitations worth discussion. The protocol prespecified that only patients with at least 3 months OAC pretreatment could be included, which limits generalizability of our findings to newly treated patients. However, this mandatory pretreatment phase was important to reduce the risk of selection bias at the time of enrollment, since OAC selection was already done as part of clinical routine and independent from the study procedures. Furthermore, thromboembolic and bleeding endpoints as well as treatment discontinuations for side effects are known to occur at a higher rate during the early phase of anticoagulant treatment.^35^^,^^36^ For newly treated patients, the need to establish a stable international normalized ratio at the beginning of VKA therapy (when subtherapeutic and supratherapeutic effects are common) would have clearly biased the study in favor of rivaroxaban. Therefore, the mandatory pretreatment phase was an important measure to reduce confounding within our study. Moreover, although data on measurements of serum creatinine or estimation of GFR were prospectively collected every 3 months during follow-up, due to the noninterventional design of the study, the data set was heterogeneous and measurements occurred inconsistently over time and could have been additionally influenced by restrictions during the COVID-19 pandemic.^37^ Thus, the power to assess outcomes such as absolute decline of eGFR over time or other established outcomes based on serum creatinine measurements such as doubling in serum creatinine or >30% decreases of eGFR was limited. On the other hand, while these are reasonable kidney outcomes to evaluate, they are only surrogate in nature. Outcomes such as the need for chronic KRT arguably carry greater clinical relevance. Regardless of the optimization of the methodology and the number of covariates used in propensity score analysis, residual confounding cannot be fully excluded.^38^ The number of patients analyzed in XARENO seems modest, however, the registry included largely patients with at least CKD stage 3b (94%) and the sample size is still comparable with the number of patients with advanced CKD that were included in other large registries.^3^^,^^22^

## Conclusions

Among patients with AF and CKD, use of rivaroxaban was associated with a reduction in patients’ risk of adverse kidney outcomes including the need for chronic KRT and a decline to an eGFR <15 mL/min/1.73 m^2^, when compared to use of VKA. This occurred against a similar risk for net clinical benefit including stroke and thromboembolism and major bleeding events. XARENO thus provides important prospective observational evidence on the effectiveness and safety of rivaroxaban and VKA therapy when used in routine practice within the vulnerable group of patients with AF and advanced CKD.

PERSPECTIVES **COMPETENCY IN PATIENT CARE AND PROCEDURAL SKILLS:** In addition to the focus on prophylaxis against thromboembolic events, particularly stroke, nephroprotection has additional clinical significance in the management of older patients with AF and advanced CKD. In this respect, OAC with rivaroxaban may offer better protection than therapy with VKA, including a reduced risk of kidney failure.

**TRANSLATIONAL OUTLOOK:** The potential benefit of treatment with DOACs such as rivaroxaban to reduce adverse kidney outcomes including kidney failure should be explored in larger prospective studies and with longer follow-up.

## Funding support and author disclosures

Funded by 10.13039/100004326Bayer AG as an investigator-initiated study. Dr Kreutz has received support for research by 10.13039/100004326Bayer AG and personal honoraria from Bayer AG, Berlin-Chemie Menarini, Daiichi Sankyo, Ferrer, Merck, Sanofi, and Servier. Dr Deray has received support for research by 10.13039/100004326Bayer AG and personal honoraria from Bayer AG. Dr Floege has received personal honoraria from AstraZeneca, Bayer AG, Boehringer, Fresenius, Novartis, and Vifor. Dr Gwechenberger has received personal honoraria from Boehringer Ingelheim, Bayer AG, and Daiichi Sankyo. Dr Hahn has received personal honoraria from Bayer AG, Daiichi Sankyo, Berlin Chemie, Vifor Pharma, AstraZeneca and financial support by AMGEN. Dr Luft has received personal honoraria from Bayer AG, Amgen, and Moleac. He has received research funding from the P&K Pühringer Foundation, The LOOP Zurich, the University of Zurich, and the Swiss National Science Foundation. Dr Persson has received personal honoraria from Bayer AG (regarding renal safety); and has received support for research on contrast media and the kidney by Bayer AG. Dr Axthelm has received institutional research support and personal honoraria from Abbott, Amgen, Astra, Bayer AG, Biotronik, Bristol Myers Squibb, Boehringer Ingelheim, Daiichi Sankyo, Esperion, Microport, Novartis, Novo Nordisk, Pfizer, Sanofi Sythelabo, and SmithKline Beecham. Dr Beer has received grant support from the Swiss National Foundation of Science, the Swiss Heart Foundation and the Stiftung Kardio; grant support, speaker and consultation fees to the institution from Bayer, Sanofi, and Daiichi Sankyo. Dr Lellouche was a speaker for and has received consulting fees from Bayer, Bristol-Myers Squibb and Pfizer. Dr Coleman has received support for research by Bayer AG, Janssen Scientific Affairs LLC, and AstraZeneca; and consulting fees from Bayer AG, Janssen Scientific Affairs LLC, and AstraZeneca. Dr Beyer-Westendorf has received institutional research support and personal honoraria from Bayer AG, Daiichi Sankyo, Pfizer, and Portola/Alexion. All other authors have reported that they have no relationships relevant to the contents of this paper to disclose.
