# Supplementary material for: Rivaroxaban vs Vitamin K Antagonist in Patients With Atrial Fibrillation and Advanced Chronic Kidney Disease
Source: JACC Adv. 2024 Jan 5;3(2):100813. doi: 10.1016/j.jacadv.2023.100813 (PMC11198259; doi:10.1016/j.jacadv.2023.100813)
Supplement: Supplementary data [file mmc1.docx]

**SUPPLEMENTAL APPENDIX**

**Supplemental Methods**

Patients treated with either rivaroxaban or a VKA were included in the registry between April 2016 and January 2020. Additionally, patients in whom physicians were withholding any anticoagulation were included in the registry (overall 89 patients) for exploratory analysis only (Figure 1). The design of the XARENO study has been reported ^1^. Accordingly, the present analysis utilized data from the rivaroxaban and VKA cohorts only ^1^.

For patients who dropped out of the study (discontinued study participation prematurely for any reason), data until the day of dropout was used for statistical analysis. Patients who switched treatment from rivaroxaban to VKA, or vice versa, remained active in the registry and data collection was continued. For patients who switched from rivaroxaban or VKA to another DOAC or any non-approved experimental anticoagulant medication an end of study visit was performed, and no additional follow-up data was collected.

The rationale for the sample size was based on a previous report ^2^ to detect a mean difference in GFR of at least 1 mL/min per 1.73m^2^ after 12 months ^1^. Additionally, the composite of blindly adjudicated categorical adverse kidney outcome including the progression to CKD stage 5, i.e. eGFR <15 mL/minute/1.73 m^2^, need for chronic kidney replacement therapy (KRT) or development of acute kidney injury (AKI) was analysed. Further, a composite net-clinical benefit outcome consisting of stroke or systemic embolism (SSE) (excluding transient ischemic attack), International Society on Thrombosis and Haemostasis (ISTH) major bleeding ^3^, myocardial infarction (MI) or acute coronary syndrome (ACS), or cardiovascular death; the composite of stroke/systemic embolism (SSE) or cardiovascular death; each component of composite outcomes individually, all-cause mortality and persistence to index (at time of enrollment) anticoagulation therapy was assessed. All outcomes were adjudicated by a central, blinded committee. The CHA_2_DS_2_-VASc score ^4^ and modified HAS-BLED score (Hypertension, Abnormal kidney/liver function, Stroke, Bleeding history or predisposition, Labile international normalized ratio, Elderly (> 65 years), Drugs/alcohol concomitantly) ^3,5^ were calculated as reported. The time-in-therapeutic range during VKA treatment was also analyzed ^6^.

To adjust for imbalances in patient characteristics between the rivaroxaban and VKA arms at baseline, we calculated propensity scores ^7^ based upon multivariable logistic regression using 42 distinct demographic, comorbidity, laboratory, and concurrent medication variables known to be risk factors for differential OAC exposure (Table 1).

Estimated propensity scores were subsequently used to weight patients for analysis using an overlap weighting approach ^8^. Accordingly, rivaroxaban patients were weighted by the probability of receiving VKA (or 1—the propensity score) and VKA patients were weighted by the probability of receiving rivaroxaban (the propensity score). Overlap weighting (OLW) was chosen as the primary method for confounder adjustment, because it allows for all eligible patients to be included in the analysis unlike propensity score matching which typically results in sample size reduction in cohorts. This was deemed important against the background of the overall modest sample size of 1455 patients. Moreover, overlap weighting has the favorable property of resulting in the exact balance (absolute standardized differences (ASDs) for all variables included in the multivariable logistic regression model used to derive propensity scores.

**Supplemental Figure 1. Study design for XARENO as reported ^1^.**


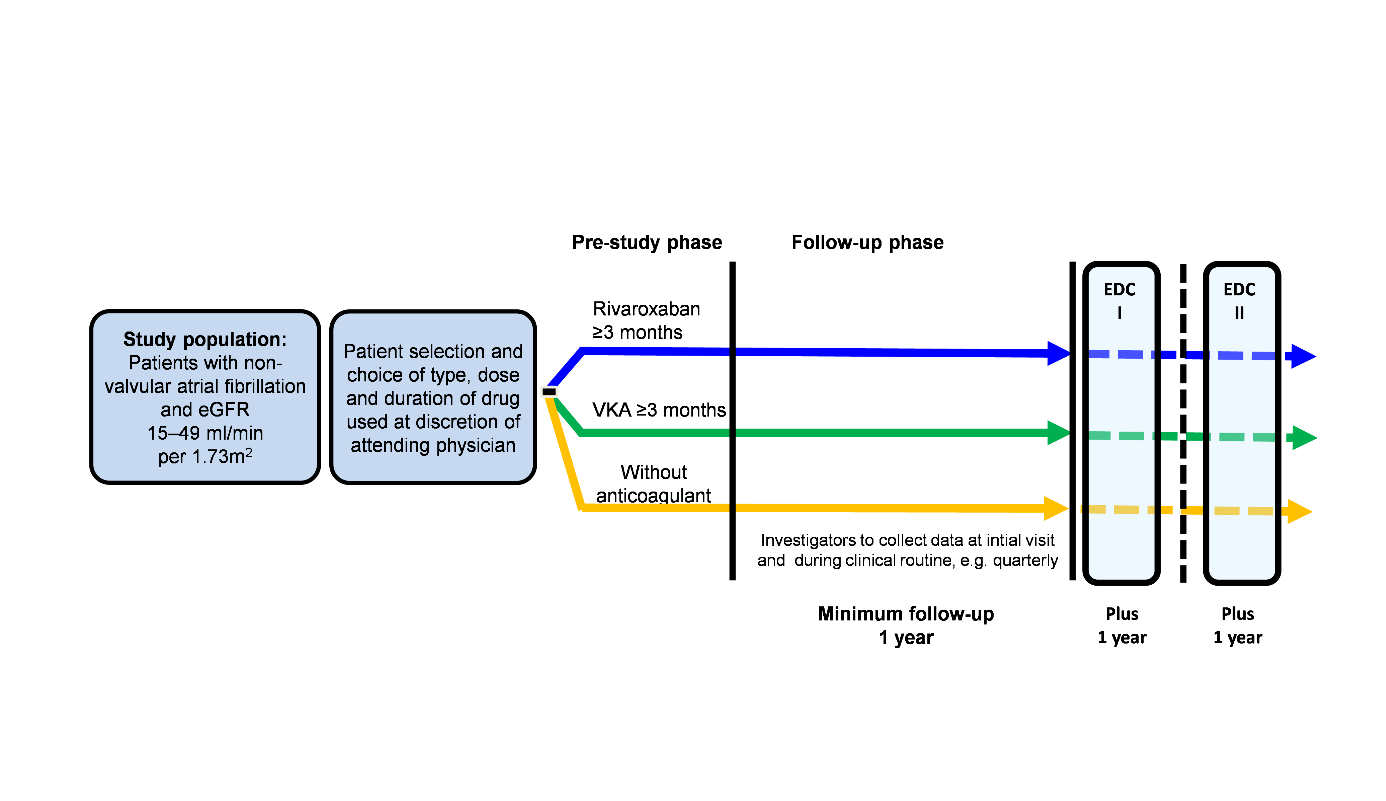
EDC = extended data collection; eGFR = estimated glomerular filtration rate; VKA = vitamin k antagonist

**References**

1. Kreutz R, Deray G, Floege J et al. Rationale and design of XARENO: XA inhibition in RENal patients with non-valvular atrial fibrillation. Observational registry. Kardiologia polska 2021;79:1265-1267.

2. Bohm M, Ezekowitz MD, Connolly SJ et al. Changes in Renal Function in Patients With Atrial Fibrillation: An Analysis From the RE-LY Trial. Journal of the American College of Cardiology 2015;65:2481-93.

3. Schulman S, Kearon C. Definition of major bleeding in clinical investigations of antihemostatic medicinal products in non-surgical patients. Journal of thrombosis and haemostasis : JTH 2005;3:692-4.

4. Hindricks G, Potpara T, Dagres N et al. 2020 ESC Guidelines for the diagnosis and management of atrial fibrillation developed in collaboration with the European Association for Cardio-Thoracic Surgery (EACTS): The Task Force for the diagnosis and management of atrial fibrillation of the European Society of Cardiology (ESC) Developed with the special contribution of the European Heart Rhythm Association (EHRA) of the ESC. European heart journal 2021;42:373-498.

5. Levey AS, Eckardt KU, Dorman NM et al. Nomenclature for kidney function and disease: report of a Kidney Disease: Improving Global Outcomes (KDIGO) Consensus Conference. Kidney international 2020;97:1117-1129.

6. Rosendaal FR, Cannegieter SC, van der Meer FJ, Briët E. A method to determine the optimal intensity of oral anticoagulant therapy. Thrombosis and haemostasis 1993;69:236-9.

7. Austin PC. An Introduction to Propensity Score Methods for Reducing the Effects of Confounding in Observational Studies. Multivariate behavioral research 2011;46:399-424.

8. Li F, Thomas LE, Li F. Addressing Extreme Propensity Scores via the Overlap Weights. American Journal of Epidemiology 2018;188:250-257.

**List of additional investigators in the XARENO registry**

Albano Laetitia (Nice, France)

Albert Catherine (Le Coudray, France)

Alexandre Joachim (Caen, France)

Al-Zoebi Ayham (Wermsdorf, Germany)

Annweiler Cedric (Angers, France)

Auer Johann (Braunau, Austria)

Balgobin Sanjeet (Cabestany, France)

Beige Joachim (Leipzig, Germany)

Berami Ahmed (Abbeville, France)

Berneau Jean-Baptiste (Bayonne, France)

Biggar Patrick (Coburg, Germany)

Birkemeyer Ralf (Ulm/Donau, Germany)

Bondke Christina (Berlin, Germany)

Bonin-Schnabel Renate (Hamburg, Germany)

Bonnemeier Hendrik (Kiel, Germany)

Bouiller Marc (Puy en Velay, France)

Boureau Anne-Sophie (Saint-Herblain, France)

Brachmann Johannes (Coburg, Germany)

Brosche Jörg (Weinböhla, Germany)

Caudmont Sebastien (Anzin, France)

Cayla Guillaume (Nimes, France)

Charpy Vianney (La Roche sur yon, France)

Constans Joel (Bordeaux, France)

Dally Jean-Baptiste (Lille, France)

De Geeter Guillaume (Anzin, France)

Debelle Fédéric (Baudour, Belgium)

Decoulx Eric (Tourcoing, France)

Delarche Nicolas (Pau, France)

Delle Karth Georg (Wien, Austria)

Delsart Pascal (Lille, France)

Derndorfer Michael (Linz, Austria)

Desprets Laurent (Cholet, France)

Dillinger Jean-Guillaume (Paris, France)

Dubart Camille (Montpellier, France)

Eberhard Katrin (Dresden, Germany)

Eichinger-Hasenauer Sabine (Wien, Austria)

Eissing Volker (Papenburg, Germany)

Erley Christiane (Berlin, Germany)

Esteve Jean-Baptiste (Caluire et Cuire, France)

Ferrari Emile (Nice, France)

Fossey-Diaz Virginie (Paris, France)

Fromentin Stéphane (Trevenans, France)

Gallouj Karim (Tourcoing, France)

Gandjbakhch Estelle (Paris, France)

Garnier Anne-Sophie (Angers, France)

Gilis Laure (Liège, Belgium)

Gondouin Bertrand (Marseille, France)

Grundmann Franziska (Köln, Germany)

Gueffet Isabelle (Nantes, France)

Haaß Sebastian (Inden-Altdorf, Germany)

Haguenhauer Didier (Colombes, France)

Hannedouche Thierry (Strasbourg, France)

Häusler Karl Georg (Berlin, Germany)

Heinz Gerd-Ulrich (Bergisch Gladbach / Bensberg, Germany)

Herold Philipp (Berlin, Germany)

Hertting Klaus (Buchholz, Germany)

Hoffer Etienne (Liège, Belgium)

Hoyer Joachim (Marburg, Germany)

Hügl Burkhard (Neuwied, Germany)

Jänsch Sybille (Dresden, Germany)

Jean-Louis Georges (Le Chesnay, France)

Jeserich Michael (Nürnberg, Germany)

Jung Werner (Villingen-Schwenningen, Germany)

Kassis Samuel (Melun, France)

Kellner Bernd-Thomas (Dornburg-Camburg)

Ketteler Marcus (Coburg, Germany)

Kielstein Jan Thomas (Braunschweig, Germany)

Koning René (Rouen, France)

Krämer Fabian (Siegen, Germany)

Krzesinski Jean-Marie (Liège, Belgium)

Lammers Ulrich (Oldenburg, Germany)

Lefebvre Jean-Marie (Lille, France)

Legrand Eric (Annonay, France)

Leschke Matthias (Esslingen, Germany)

Lodde Bernhard-Paul (Dortmund, Germany)

Maalouli Christian (Tournai, Belgium)

Mahnkopf Christian (Coburg, Germany)

Mailliez Sebastien (Abbeville, France)

Mansourati Jacques (Brest, France)

Marijon Eloi (Paris, France)

Meyer Christian (Hamburg, Germany)

Moll Detlev (Remscheid, Germany)

Montalescot Gilles (Paris, France)

Motte Serge (Bruxelles, Belgium)

Mouquet Vincent (Abbeville, France)

Nedeltchev Krassen (Aarau, Switzerland)

Neykova Anna (Thonon, France)

Nothroff Jörg (Burg, Germany)

Poyet Raphael (Toulon, France)

Prondzinsky Roland (Merseburg, Germany)

Rauch-Kröhnert Ursula (Berlin, Germany)

Richard Frank (Erfurt, Germany)

Rieker Werner (Berlin, Germany)

Rocco Andrea (Berlin, Germany)

Rostock Thomas (Mainz, Germany)

Scherr Daniel (Graz, Austria)

Schlitt Axel (Quedlinburg, Germany)

Schmidt-Gürtler Hans (Hannover, Germany)

Schön Norbert (Mühldorf am Inn, Germany)

Schwab Johannes (Nürnberg, Germany)

Schwencke Carsten (Hamburg, Germany)

Schwimmbeck Peter (Leverkusen, Germany)

Schwinger Robert H. G. (Weiden, Germany)

Schwittay Andreas (Böhlen, Germany)

Sibon Igor (Bordeaux, France)

Spengler Ulrike (Borsdorf, Germany)

Stadelmann Alexander (Nürnberg, Germany)

Steinwender Clemens (Linz, Austria)

Stöhring Reinhard (Bad Homburg, Germany)

Stolear Jean-Claude (Tournai, Belgium)

Taldir Guillaume (Saint Brieuc, France)

Tartière Jean-Michel (Toulon, France)

Treille Serge (Charleroi, Belgium)

Tremolieres Pierre (Montpellier, France)

Tubail Zead (Metz, France)

Warling Xavier (Liège, Belgium)

Wetzstein Morgane (Abbeville, France)

Zaman Adrian (Kiel, Germany)

Zemmrich Claudia (Berlin, Germany)
